# Investigation of potential genotoxic activity using the SOS Chromotest for real paracetamol wastewater and the wastewater treated by the Fenton process

**DOI:** 10.1186/s40201-015-0220-0

**Published:** 2015-09-29

**Authors:** Emel Kocak

**Affiliations:** Department of Environmental Engineering, Faculty of Civil Engineering, Yildiz Technical University, Istanbul, Turkey

**Keywords:** Induction factor, *Escherichia coli* PQ37, Fenton process, Real paracetamol wastewater, SOS Chromotest

## Abstract

**Background:**

The potential genotoxic activity associated with high strength real paracetamol (PCT) wastewater (COD = 40,000 mg/L, TOC = 12,000 mg/L, BOD_5_ = 19,320 mg/L) from a large-scale drug-producing plant in the Marmara Region, was investigated in pre- and post- treated wastewater by the Fenton process (COD = 2,920 mg/L, TOC = 880 mg/L; BOD_5_ = 870 mg/L).

**Methods:**

The SOS Chromotest, which is based on *Escherichia coli* PQ37 activities, was used for the assessment of genotoxicity. The corrected induction factors (CIF) values used as quantitative measurements of the genotoxic activity were obtained from a total of four different dilutions (100, 50, 6.25, and 0.078 % v/v.) for two samples, in triplicate, to detect potentially genotoxic activities with the SOS Chromotest.

**Results:**

The results of the SOS Chromotest demonstrated CIF_max_ value of 1.24, indicating that the PCT effluent (non-treated) is genotoxic. The results of the SOS Chromotest showed an CIF_max_ value of 1.72, indicating that the wastewater treated by Fenton process is genotoxic.

**Conclusions:**

The findings of this study clearly reveal that the PCT wastewater (non-treated) samples have a potentially hazardous impact on the aquatic environment before treatment, and in the wastewater that was treated by the Fenton process, genotoxicity generally increased.

## Introduction

Pharmaceutical drugs can reach the aquatic environment from domestic waste or industrial wastewater, hospitals, and health care centers [1]. Considering the complex nature of a large variety of pharmaceutical drugs, genotoxicologic studies of real paracetamol wastewater remained very superficial, and therefore it is necessary to further examine assay systems that have the ability to evaluate the substantial impact of some of the more persistent pollution sources. Unused drugs, manufacturing waste, and sewage sludge can also be introduced to the environment by way of landfill leachates [2].

Over the past few years, advanced oxidation processes (AOPs) are used to reduce contamination based on the presence of stable pharmaceuticals [3–6]. Complex organic chemicals are formed during the production of pharmaceuticals and it is not easy to remove these compounds biologically. As a result, AOPs are more appropriate than conventional methods to treat pharmaceutical wastewater [7]. AOPs comprises Fenton, photo-Fenton, and ozonation combined with UV-light and/or H_2_O_2_, mainly TiO_2_-mediated photocatalysis [6], electrolysis, wet air oxidation, ultrasound and ionizing radiation, microwaves, pulsed plasma, and the ferrate reagent [5]. Different reviews of the literature have reported the fate of some pharmaceutical compounds as well as their occurrence and effects in the aquatic environment [8, 9]. Some of the substances found in wastewater are genotoxic and are suspected to be a possible cause of the cancers observed in previous decades. Water genotoxicity studies are of interest because epidemiologic investigations have shown a link between genotoxic drinking water intake and a rise in cancer [10–12]. The results of these studies must however, be interpreted with caution because the exposure to genotoxic water was only estimated and not truly measured. However, these results emphasized the importance of the determination of water genotoxicity with an aim of controlling the population exposure. Thus, the monitoring of water contamination for potentially carcinogenic compounds represents a major concern for human health [13]. It is extremely difficult to quantify the risk associated with these chemical pollutants because they usually occur in concentrations too low to allow analytical determination, and putative mutagens, with few exceptions, have never ever been identified. Moreover, the composite effects of mixtures cannot be readily assessed via analytical methods. Thus, toxicity is often evaluated by means of biological tests, as well as by bacterial genotoxicity tests which do not require a priori knowledge of toxicant identity and/or physical-chemical properties [14].

To the best of the authors’ knowledge, there are no studies dealing with the high strength real paracetamol (PCT) wastewater genotoxicity by way of the SOS Chromotest. However, all hospital wastewater studies show that this kind of wastewater including drugs and antibiotics could have a genotoxic potential [15–18]. Genotoxicity was studied with the SOS Chromotest, which allows for the detection of primary DNA damaging agents in *Escherichia coli*. Based on the above-mentioned facts, the specific objectives of the present study were as follows: (1) to evaluate the main characteristics of the real PCT wastewater, (2) to use the SOS Chromotest microplate assay to investigate the genotoxic activities of the non-treated PCT wastewater, (3) to use the SOS Chromotest microplate assay to investigate the genotoxic activities of the wastewater treated by the Fenton process.

## Materials and methods

### Characteristics of the PCT wastewater

High COD values of real PCT wastewater are related to the high concentrations of PCT, PAP, and aniline. Pollutant concentrations of the wastewater can change day by day according to the process operations [19]. In this study, wastewater with a COD concentration of 40,000 mg/L was used for the investigation of potentially genotoxic activity with the SOS Chromotest. The main characteristics of real PCT wastewater are shown in Table 1 [19].

**Table 1:** Main characteristics of real paracetamol (PCT) wastewater [19]

Parameter	Value
pH	9.0
Chemical oxygen demand, COD (mg/L)	40,000
5-day biological oxygen demand, BOD_5_ (mg/L)	19,320
Total organic carbon, TOC (mg/L)	12,000
Paracetamol, PCT (ppm)	107
Para-amino phenol, PAP (ppm)	1818
Aniline (ppm)	2915

As seen in Table 1, pollutant concentrations are extremely high in PCT wastewater and consequently, the treatability of this wastewater is very difficult in conventional treatment plants. As the wastewater contains different chemicals, the presence of the toxic effect derived from chemical products that could not be removed through conventional biological treatment methods, and also the low BOD/COD ratio show that the wastewater must be pretreated chemically. Badawy et al. [20] indicated that wastewater with a BOD/COD ratio between 0.25 and 0.30 cannot be treated biologically.

### Characteristics of wastewater treated by the Fenton process

The aim of the Fenton process, which has been previously studied, is obtaining the highest COD removal efficiency using the optimum chemical dosages. In this study, wastewater treated by the Fenton process was used as the second sample to investigate potential genotoxic activity with the SOS Chromotest. Characteristics of the wastewater treated by the Fenton process are shown in Table 2 [19].

**Table 2:** Characteristics of the wastewater treated by the Fenton process [19]

Parameter	Value
pH	6.0
Chemical oxygen demand, COD (mg/L)	2920
5-day biological oxygen demand, BOD_5_ (mg/L)	870
Total organic carbon, TOC (mg/L)	880
Paracetamol, PCT (ppm)	1
Para-amino phenol, PAP (ppm)	2
Aniline (ppm)	17

### SOS Chromotest

The SOS Chromotest is a colorimetric assay of the enzymatic activities that occur after incubating the test strain of bacteria in the presence of various amounts of experimental samples [21]. The test utilizes a genetically engineered bacterium, *E. coli* PQ37, to detect DNA-damaging agents. In this assay, the *β-galactosidase* (*β -gal*) gene (*lacZ*) of the *E. coli* PQ37 tester strain is fused to the bacterial *sfiA* SOS operon. Thus, *lacZ* is concomitantly expressed during the bacterial SOS response, and this gene expression can be photometrically determined by the induction of *β-gal*. The amount of *β-gal* induction is indicative of the extent of SOS induction and bacterial genotoxicity. Bacterial *alkaline phosphatase* (*AP*) activity was used to determine the range of bacterial cytotoxicity. The ratio of *β-galAP* activity was defined as the induction factor (IF), and this ratio was used to indicate the extent of SOS induction for the tested compounds [20]. The test is available as a test kit, which includes all of the necessary materials. No special measuring devices, with the exception of a plate reader, were required to complete this assay. This test can also be used as a qualitative test, based on the use of a color scale. The assay can be completed within 24 h, including the revival of the bacteria. The test detects any primary DNA damage that is caused by genotoxins, and the test can be used for various types of aqueous samples.

In this study, the SOS Chromotest was performed, without metabolic activation, as described by Quillardet and Hofnung [22]. The *E. coli* PQ37 tester strain was kindly provided by Environmental Bio-Detection Products Inc. [21]. Four different dilutions (100, 50, 6.25, and 0.078 % v/v.) for two samples, in triplicate, and the testing began with a 100 mL sample that was equal for each cuvette. The test was performed at 37 °C, and the cuvettes were read after 2 h with a spectrophotometer. Spectrophotometer equipped with 600 nm filter and using 1 cm light-path rectangular cuvettes (for preparation of the bacterial suspension). Growth bacteria suspension was required OD of 0.05 at 600 nm by the spectrophotometer before use in the assay depending upon the degree of growth obtained. The bacteria was grown in 37 °C, incubator to an OD (optical density) of 0.05 to 0.06 in approximately 4 h and the test was run. When this method was used the bacteria were still in log phase growth and the colour development, when exposed to a genotoxin, would have occured within an hour or so. If the OD was is 0.05 colour development would have taken approximately 1.5 h. If the OD was closer to 0.07 the colour development would have occurred within half and hour because of the increased cell density [21, 23].

For the direct assay, the negative control was composed of a 10 % DMSO (dimethyl sulfoxide) solution in sterile, ultrapure water, and the positive control was 4-nitro-quinolineoxide (4NQO).

### Determination of genotoxic activity

The SOS Chromotest involves incubating the bacteria with the experimental sample and assessing the *β-galactosidase* (*β-gal*) activity (i.e. the level of SOS induction). *Alkaline phosphatase* (*AP*) activity is also measured and serves as a control for toxicity [15]. *AP* reduction factors (RF), *β-gal* induction factors (IF), and corrected induction factors (CIF = IF/RF) were calculated as described by Legault [24].

1 $$ RF=\frac{{\left(O{D}_{405}\right)}_{mean,t}}{{\left(O{D}_{405}\right)}_{mean,c}} $$

2 $$ IF=\frac{{\left(O{D}_{620}\right)}_{mean,t}}{{\left(O{D}_{620}\right)}_{mean,c}} $$

3 $$ CIF=\frac{RF}{IF} $$

where (OD_405_) mean and (OD_620_) mean are the means of the optical density (OD) readings that were taken at 620 nm (*β-gal*) and 405 nm (*AP*), and *t* and *c* refer to the test and the control dilutions, respectively. Bombardier et al. [25] reported that the RF and IF values account for the background activity of the control. The ratio of IF to RF units yields an estimate of *β-gal* activity that is corrected for toxicity. The criterion that was used to consider a sample as “positive” in the SOS Chromotest differs between authors [13, 24, 26, 27]. In the present study, significant genotoxic activity was defined as having a corrected induction factor that was equal to or greater than 1.2–1.5, as suggested by most of the previously published studies [13, 24, 28].

All SOS Chromotest analyses were conducted according to the EBPI (Environmental Bio-Detection Products Inc.) protocols [21]. The experimental equipment (i.e. micropipettes, Eppendorf pipettes) were autoclave sterilized at 121 °C and 10.6 bar for 15 min (Nuve OT 032). A minishaker with an orbit of 4.5 mm and a speed range of 0–2500 rpm (IKA Labortechnik, Staufen, Germany) was used to centrifuge the bacteria at a fixed agitation speed of 1500 rpm. The bacteria were grown at a stable temperature of 37 °C in a temperature-controlled incubator (Memmert, Germany). This incubator was also used for the development of the enzymatic activities. The bacteria cultures were grown, and the optical density values (600 nm) were measured using an UV–VIS Spectrophotometer (Shimadzu, UV-1202, UV–VIS) with a special quartz cuvette that allowed for a light path length of 1 cm. ATP and b-gal activities were measured, using a Biotek PowerWave XS Microplate ELISA Reader (BioTek Instruments Inc., Winooski, VT, USA) with data analysis software (Gen 5), at 405 nm (OD_405_) and 620 nm (OD_620_), respectively. The pH values of the samples were measured with a pH meter (Jenway 3040 Ion Analyzer) and a pH probe (HI1230, Hanna Instruments, USA). Deionized and sterile ultrapure water was used in the experiments, and the water was supplied from a TKA-GenPure water purification system (Niederelbert, Germany). The physicochemical analyses of the surface water samples were conducted by the procedures described in the Standard Methods [23, 29].

## Results and discussion

In the present study, the SOS Chromotest based on *Escherichia coli* PQ37 activities was used for the assessment of genotoxicity of samples of real PCT wastewater before and after the wastewater was treated by the Fenton process. SOS responses were determined as corrected induction factors (CIF) for all samples and presented in Table 3. The tests demonstrate that these PCT samples pre- and post-treatment present a genotoxic effect. Indeed, out of a total of eight samples tested, four were positive (50 %). It is difficult to compare these results with other studies because many parameters can influence the genotoxicity test response (composition of the samples, nature of paracetamol, nature of chemicals used in the Fenton process, nature of the genotoxicity test, etc.) [13].

**Table 3:** Results of the SOS Chromotest on the PCT wastewater pre- and post-treatment by the Fenton process

Sample name	V (%)	CIF	SD
Real PCT wastewater (non-treated)	100	0,64	0,07
	50	**1,24**	0,03
	6,25	0,84	0,05
	0,078	0,92	0,24
PCT wastewater treated by the Fenton proces	100	**1,50**	0,04
	50	**1,72**	0,29
	6,25	**1,25**	0,04
	0,078	0,99	0,15

V, tested concentration; CIF, corrected induction factor; SD, standard deviation

Genotoxic samples are indicated in bold letters

An appraisal of the genotoxicity of the PCT samples before and after treatment is as follows: (a) for the real PCT wastewater: of a total of four samples tested, one was positive that CIF = 1.24 (25 %), and (b) for the PCT wastewater treated by the Fenton process: of a total of four samples tested, three were positive that CIF = 1.25, 1.50, and 1.72 (75 %). Some of the calculated CIF values were determined to be above the level that is considered to be the 1.2 threshold level. The CIF values of all the PCT wastewater samples (non-treated and treated by Fenton process) were observed within the 1.24–1.72 range. When the CIF for any of the test concentrations reached 1.2, the test substance was scored as significantly genotoxic. However, the SOS Chromotest results clearly indicated that genotoxic effects that were found in the PCT wastewater (non-treated and treated by the Fenton process) samples. Table 4 summarizes the genotoxic activity levels and the corresponding threshold values that were defined in the different studies. In the present study, significant genotoxic activity was defined as having a corrected induction factor that was equal to or greater than 1.2, as suggested by the published studies authors [13, 14, 24, 28, 30].

**Table 4:** Genotoxic activity levels and the corresponding threshold values defined in different studies

Genotoxic activity levels	Corrected induction factors (CIF)	References and region
SOS response	>1.2	Legault et al. (1996), Canada
Genotoxic	>1.2	Kocak et al. (2010), Turkey
β-galactosidase activity significantly increases compared with the solvent control	>1.5	Jolibois and Guerbet (2005), France
Genotoxic	>1.5	Mersch-Sundermann et al. (1992), Germany
Genotoxic	>1.5	Margulis et al. (2003), Russia

The performance data revealed that a wide range of CIF values were observed, and the range of CIF values depends on the characteristics of wastewater matrices of PCT and chemical dosage for the Fenton process. High genotoxic activity values are probably due to the presence of several mutagenic and carcinogenic agents, which include persistent components, soluble DNA-damaging products, recalcitrant substances, and other undesirable impurities that are present in the wastewater samples. It is apparent from previous studies that various chemical compounds have been widely used in numerous industrial and environmental applications. However, relatively few genotoxicological investigations are available in the literature. Therefore, additional studies that use genotoxicological data, in addition to the contaminant monitoring data, will be necessary to identify the sources of the toxicants and to ensure that more environmental risk assessments can be verified. Both of these points have been suggested by other researchers [13, 31].

## Conclusions

This study showed that the real PCT wastewater before and after treatment are genotoxic. Especially after the Fenton process, genotoxicity generally increased. As a consequence of the different chemical species present in the paracetamol wastewater, the Fenton process was able to increase wastewater genotoxicity; especially after the Fenton process, genotoxicity generally increased. The success of this assay was, at least in part, due to its simplicity and rapidity. The SOS Chromotest responses clearly indicated that there were potential genotoxic impacts, in terms of CIF values, found in the PCT wastewater. Some of the calculated CIF values were determined to be above the 1.2 threshold level. During the PCT process, the CIF variations were much lower than CIF variations that were observed during the Fenton process. These variations possibly depend on the chemical dosing during the Fenton process.

It is noted that the work described here is the first report from an integrated study investigating genotoxicity on PCT (non-treated and treated with Fenton process) wastewater. Although the SOS Chromotest responses indicated that the PCT wastewater was found to have genotoxic effects on the aquatic environment, further investigations will be conducted on other in vitro tests to better characterize the genotoxicity responses. This study can provide useful information to medical and water managers and health authorities in evaluation of water quality strategies for reduction of genotoxic compounds in the PCT wastewater before and after treatment.
